# Network pharmacology and *in silico* analysis reveal Kochiae Fructus as a potential therapeutic against atopic dermatitis through immunomodulatory pathway interactions

**DOI:** 10.1371/journal.pone.0320818

**Published:** 2025-04-03

**Authors:** Shakeel Ahmad Khan

**Affiliations:** 1 Department of Chemistry, University of Management and Technology Lahore, Pakistan; 2 Department of Applied Biology and Chemical Technology, The Hong Kong Polytechnic University, Kowloon, Hong Kong SAR, China; Saveetha University - Poonamallee Campus: SIMATS Deemed University, India

## Abstract

Atopic dermatitis (AD), a chronic inflammatory disorder, poses significant therapeutic challenges owing to its complex pathophysiology, involving disrupted epidermal barrier function and immune dysregulation. This study investigated the therapeutic potential of Kochiae Fructus in AD treatment using bioinformatics, including network pharmacology and molecular docking techniques. We identified 19 key phytochemicals from Kochiae Fructus and 268 potential targets using the Traditional Chinese Medicine Systems Pharmacology Database (TCMSP) and SwissTarget Prediction. Using GeneCards, 1786 AD-related genes were retrieved, resulting in 116 intersecting gene targets for further analysis. Protein-protein interaction (PPI) networks and Molecular Complex Detection (MCODE) analyses highlighted 78 anti-AD key targets, including SRC, MAPK3, MAPK1, JUN, PIK3CA, ESR1, PTGS2, PTPN11, IL-6, and ALOX5, among the top ten anti-AD core targets. Gene ontology (GO) enrichment analysis revealed that Kochiae Fructus affects biological processes and molecular functions, such as positive regulation of the apoptotic response, inflammatory response, and hormone-mediated signaling pathways, which may be associated with its anti-AD effects. Kyoto Encyclopedia of Genes and Genomes (KEGG) pathway analysis showed that the C-type lectin receptor signaling pathway is the main pathway involved in the anti-AD effects of Kochiae Fructus, which interacts with a notably larger number of anti-AD core targets and plays a direct role in intensifying crucial inflammatory and immune responses in the heart of AD pathogenesis. Molecular docking demonstrated robust binding affinities of key phytochemicals, particularly ecdysterone and 11,14-eicosadienoic acid, to the anti-AD core targets. Molecular dynamics simulations of over 1000 ns confirmed the stability and potential efficacy of these interactions. Hence, this study underscores the therapeutic potential of Kochiae Fructus in AD management, offering a mechanistic basis for its clinical application and paving the way for novel anti-AD strategies that leverage TCM phytochemicals.

## 1. Introduction

Atopic dermatitis (AD) is a pervasive inflammatory dermatological condition characterized by compromised epidermal barrier function and diminished moisture retention capacity [1,2]. This pathology is characterized by clinical manifestations including pruritus [3], xerosis, fissuring [4], erythema [5], lichenification [6], and cutaneous inflammation [7], concomitant with keratinocyte apoptosis [8]. Epidemiological evidence delineates a progressive escalation in AD prevalence, affecting up to 20% of the pediatric population [9] and up to 10% of adults globally [10], positioning it as the 15^th^ most common nonfatal dermatological disorder [1, 10–12]. AD exhibits a predilection for early onset, frequently identified during infancy and early childhood, with potential persistence into adulthood or an initial manifestation in later life stages. Notably, prevalence rates exhibit variability across demographic groups, influenced by racial and ethnic distinctions. In the United States, for instance, a higher incidence was observed in African American children (19.3%) than in their Caucasian counterparts (16.1%) [1, 12,13]. Similarly, in Hong Kong, the prevalence is approximately 20% across the lifespan, with a significant pediatric burden affecting approximately 30% of children [14–16]. The etiological attribution of the rising AD incidence has been tentatively linked to environmental factors, particularly in affluent and industrialized locales, where exposure to air pollutants and domestic hygiene products prevail [1]. This correlation necessitates a comprehensive etiological exploration to unravel the complexities of AD and to develop new targeted therapeutic and prophylactic strategies [17,18].

Pathogenetically, AD is ascribed to disruption of the integrity of the epidermal barrier, which facilitates allergenic penetration and subsequent immunologic activation [1]. This disruption triggers the activation of dendritic and innate lymphoid cells, which subsequently recruit and activate Th2 lymphocytes. The activation of these lymphocytes leads to the dysregulated secretion of pro-inflammatory cytokines, such as interleukin-4 (IL-4), interleukin-13 (IL-13), and interleukin-31 (IL-31), which precipitate the inflammatory cascade in AD through the Janus kinase (JAK) signaling pathways. This cascade further propagates inflammation and AD pathology by activating plasma cells and B lymphocytes, thereby inducing the production of antigen-specific immunoglobulin E (IgE) and amplifying the inflammatory response [1,18–21].

The therapeutic landscape for AD, devoid of a definitive cure, is predominantly anchored by the use of topical anti-inflammatory agents such as corticosteroids and calcineurin inhibitors [18,22]. While corticosteroids have proven effective in alleviating AD symptoms, their chronic use is marred by potential adverse effects, including irreversible skin atrophy and systemic repercussions, such as adrenal suppression [18,23]. Calcineurin inhibitors, such as tacrolimus and pimecrolimus, offer symptomatic relief by inhibiting the transcription of critical cytokines (IL-2, IL-4, and IL-5) involved in AD pathogenesis, albeit with lingering concerns regarding their oncogenic potential [18, 24–26]. In this therapeutic milieu, Traditional Chinese Medicine (TCM) has emerged as a noteworthy alternative that offers immunomodulatory benefits for chronic conditions with a favorable safety profile [18].

Kochiae Fructus, the desiccated fruit of Kochia scoparia (L.) Schrad., revered as a “top grade” herb in the seminal “Shennong Ben Cao Jing,” has been a staple in TCM for millennia [27,28]. Its therapeutic applications, documented in historical pharmacopeias such as the Herbal Canon and Compendium of Materia Medica [28,29], encompass a range of conditions, including eczema [28], dermatitis [29,30], pruritus [3], and psoriasis [31]. Phytochemical investigations have identified over 150 compounds in Kochiae Fructus, encompassing a diverse phytochemical spectrum with demonstrated pharmacological activities, including anticancer, antimicrobial, anti-edema, anti-allergic, anti-inflammatory, anti-dermatitic, anti-pruritogenic, and hypoglycemic effects [28].

**Fig 1:**
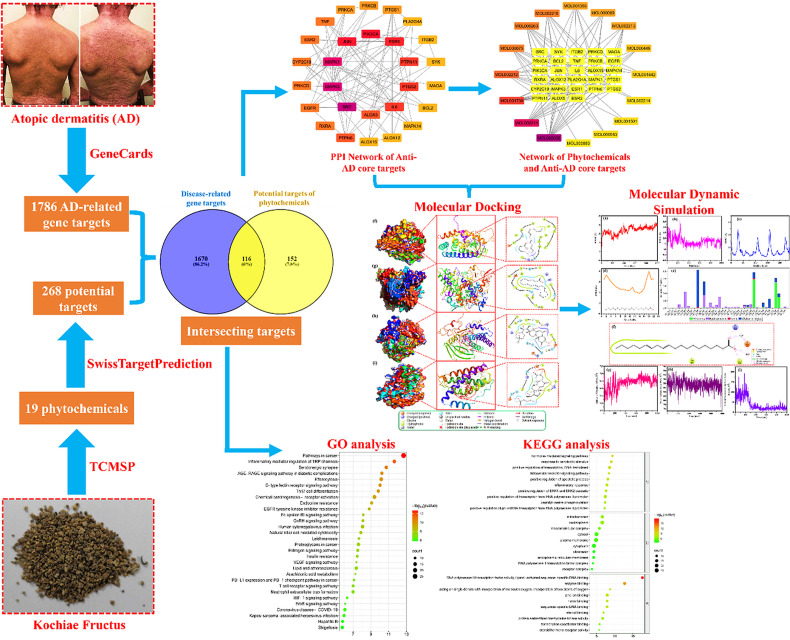
Schematic workflow of the current study. Patients with severe AD and Kochiae Fructus images were taken from [28,36], respectively under Creative Commons licenses.

Contemporary TCM practices often incorporate Kochiae Fructus into polyherbal formulations for AD treatment [32–34]. However, the specific phytochemicals and their mechanistic pathways in AD therapy remain elusive. In light of these gaps, the application of bioinformatics, a burgeoning field intertwining big data and artificial intelligence, holds promise for identifying active pharmaceutical ingredients and elucidating underlying mechanisms of drugs [35]. Thus, this study leveraged bioinformatics methodologies such as network pharmacology and molecular docking to decipher the biological phytochemicals, molecular targets, and pathways integral to the anti-AD effects of Kochiae Fructus. Fig 1 shows a schematic workflow of the current study.

## 2. Materials and methods

### 2.1. Software/databases

Network pharmacology for the phytochemicals of Kochiae Fructus, its common targets, and AD targets was accomplished using different web tools and software (Table 1).

**Table 1:** Software and web tools used for network pharmacology studies.

Sr. No.	Name	Online Link	References
1	TCMSP (Version 2.3)	https://old.tcmsp-e.com/tcmsp.php	[37]
2	GeneCards^®^: The Human Gene Database	https://www.genecards.org/	[38]
3	Venny 2.1	https://bioinfogp.cnb.csic.es/tools/venny/	[39]
4	SwissTargetPrediction	http://www.swisstargetprediction.ch/	[40]
5	STRING (version 11.5)	https://string-db.org/	[41]
6	Cytoscape (version 3.9.0)	https://Cytoscape.org/	[42]
7	Cytoscape’s Molecular Complex Detection (MCODE) plug-in	ftp://ftp.mshri.on.ca/pub/BIND/Tools/MCODE	[42]
8	DAVID (Version 6.8)	https://david.ncifcrf.gov/	[43]
9	Bioinformatics platform	http://www.bioinformatics.com.cn/	[44]
10	Protein Data Bank	https://www.rcsb.org/	[45]
11	NCBI PubChem	https://pubchem.ncbi.nlm.nih.gov/	[46]
13	PyMOL	https://pymol.org/support.html	[47]
15	Schrödinger Suite	https://www.schrodinger.com/	[48–50]
16	Desmond	https://www.deshawresearch.com/research.html	[51]

### 2.2. Retrieval of phytochemicals from Kochiae Fructus

The phytochemicals of Kochiae Fructus were retrieved from the Traditional Chinese Medicine Systems Pharmacology Database (TCMSP, Version 2.3, available at https://old.tcmsp-e.com/tcmsp.php, accessed on March 10, 2024) [37]. A total of 19 phytochemicals were identified and are listed in S1 Table.

### 2.3. SwissTargetPrediction for phytochemicals of Kochiae Fructus

The SwissTargetPrediction database (http://www.swisstargetprediction.ch/, accessed on March 10, 2024) was used to ascertain the potential biological targets of Kochiae Fructus phytochemicals [40]. Only targets with a probability score of>  zero were earmarked for further examination.

### 2.4. Identification of atopic dermatitis-related genes

Genes implicated in AD were extracted from GeneCards^®^: The Human Gene Database (accessible at https://www.genecards.org/, accessed on March 10, 2024) using search terms such as “atopic dermatitis,” “atopic dermatitis inhibiting,” and “anti-atopic dermatitis” [38]. Only AD-related genes with a relevance score of ≥  5 were earmarked for further analysis [52].

### 2.5. Delineation of intersection gene targets

The confluence of gene targets implicated in both the potential targets of Kochiae Fructus’s phytochemicals and the identified AD-related genes was ascertained using Venny 2.1, an online software tool (https://bioinfogp.cnb.csic.es/tools/venny/; accessed on March 10, 2024) [39].

### 2.6. Protein-protein interaction (PPI) analysis

Protein-protein interaction networks for the intersecting gene targets were generated using the STRING database (https://string-db.org/, version 12.0, accessed on March 10, 2024), with the highest confidence score threshold (0.900), restricted to Homo sapiens, [18,41]and the disconnected nodes were removed from the network. Subsequently, the PPI network from STRING in the form of tab-separated values (tsv.) file format was imported into Cytoscape software (version 3.10.1) [42]. Subsequent topological analyses of network mapping were performed using the analyze network option in the Cytoscape software tool, which facilitated the identification of anti-AD core targets based on their degree centrality (DC) values. The identified anti-AD core targets were further verified using the CytoHubba plug-in in the Cytoscape software, and the top 10 anti-AD core targets with the largest DC values were ranked out and further used for subsequent molecular docking.

### 2.7. Molecular Complex Detection (MCODE) analysis

The critical modules in the PPI network of 78 potential anti-AD key targets were identified using the Cystoscope MCODE plug-in [42]. The conditions for MCODE analysis were as follows: find clusters in the whole network, degree cutoff =  2, node score cutoff =  0.2, k-core =  2, and Maximum Depth =  100 [53].

### 2.8. Network construction between AD-related genes and phytochemicals

An integrated network analysis encompassing intersecting AD-related genes and phytochemicals of Kochiae Fructus was conducted using the Cytoscape software (version 3.10.1) [42] to elucidate the complex interplay and potential therapeutic pathways.

### 2.9. Enrichment analysis

Gene Ontology (GO) functional annotation and Kyoto Encyclopedia of Genes and Genomes (KEGG) pathway analyses were performed on 78 intersecting gene targets using DAVID (version 6.8, available at https://david.ncifcrf.gov/, accessed on March 10, 2024) [54,55]. GO terms were categorized into cellular components (CC), biological processes (BP), and molecular functions (MF). The top-ranked terms and pathways were visualized as an enrichment dot bubble chart, employing a bioinformatics platform for a comprehensive presentation. The classical hypergeometric test, adjusted for multiple hypothesis testing using the Benjamini–Hochberg method, was applied to ascertain statistical significance, with an adjusted p-value threshold of less than 0.05 [18].

### 2.10. Molecular docking

Maestro 13.5, from the Schrödinger Suite 2023 version 1, was utilized for the preparation of proteins and ligands [56]. Glide within the same suite was used for molecular docking, whereas Desmond-2020 version 1 facilitated molecular dynamic (MD) simulation studies [51,56].

#### 2.10.1. Anti-AD core targets: preparation.

The top ten potential anti-AD core targets (SRC, MAPK3, MAPK1, JUN, PIK3CA, ESR1, PTGS2, PTPN11, IL-6, and ALOX5), represented by PDB IDs (1O46, 4QTB, 4FUX, 1A02, 6VO7, 1XP1, 5KIR, 4RDD, 1IL6, and 3V92) respectively, were retrieved from the Protein Data Bank (PDB) database (https://www.rcsb.org/). These protein structures were imported into the Protein Preparation Wizard (PPW) of the Schrödinger Suite 2023-1 with several modifications [50]. These modifications include the addition of hydrogen, assignment of bond orders, optimization of hydrogen positions, and correction of side chains. Energy minimization was strategically performed to maintain the rigidity of the backbone atoms while allowing the flexibility of the side chains [57].

The procedure corrected missing structural elements, including loops and disulfide bonds, with the pH settings adjusted to 7.4 to mirror physiological conditions. The Prime module of the Schrödinger suite was employed to address missing side chains. Water molecules positioned further than 5 Å were removed and the metals were modeled with zero-order bonds. A secondary water removal step ensured that the remaining water molecules were at least 3 Å away from the hydrogen atoms of the non-water entities. The OPLS_2005 force field, alongside an optimization algorithm, was utilized for further energy minimization, with a heavy atom root mean square deviation (RMSD) threshold of 0.30 Å, ensuring the refinement process achieved the desired level of precision [51,57,58].

#### 2.10.2. Ligand preparation.

The ligands MOL002038 (9E,12Z-octadecadienoic acid), MOL002211 (11,14-eicosadienoic acid), MOL001739 (Zoomaric acid), MOL002212 (Ecdysterone), MOL000263 (Oleanolic acid), MOL000675 (Oleic acid), and MOL002215 (Oleanic acid) were sourced from the TCMSP database in.mol2 file format [37]. The stabilization and preparation of these ligand structures were meticulously conducted using the LigPrep module of the Schrödinger suite 2023-1 [49]. During this process, 32 stereoisomers, various ionization states, and tautomeric forms were generated at pH 7.4 ±  2.0, for each ligand to ensure comprehensive representation. The preparation steps included the addition of hydrogen atoms, energy minimization, and the application of an appropriate force field (OPLS_2005) to refine their three-dimensional (3D) coordinates and optimize them for subsequent computational analyses [57,59].

#### 2.10.3. Receptor grid generation.

The receptor grid generation tool within the Schrödinger Suite 2023-1 was employed to construct a receptor grid with grid resolution, center coordinates, and dimensions derived from this utility [48]. This process delineates the active site in proximity to the specified coordinates (x, y, z), with grid dimensions tailored to the active site ligands (reference ligands) of particular proteins. In cases where proteins lacked ligands in their active sites, the potential binding surfaces were predicted using the Sitemap function of the Schrödinger Suite 2023-1. This function not only predicts binding sites, but also provides a sitemap score, offering detailed insights into the binding site with clearly delineated regions. The utilization of the Sitemap results facilitated the identification of potential alternative binding sites or the validation of known binding sites. The grid details are listed in S2 Table.

#### 2.10.4. Glide docking of prepared Anti-AD core targets and ligands.

The Glide module of the Schrödinger Suite 2023-1 was utilized to perform molecular docking between the ten pre-prepared anti-AD core targets and the seven key phytochemicals from Kochiae Fructus [48]. Glide facilitates three docking precision modes with rigid receptor docking: high-throughput virtual screening (HTVS), standard precision (SP), and extra precision (XP). For this study, extra precision (XP) docking was selected following the upload of the receptor grid file for the respective anti-AD core targets, with all additional parameters and scoring functions maintained at their default configurations. The parameter set for each docking execution included the number of generated poses and chosen sampling methods [60]. The initial docking computation yielded various ligand poses within the binding site, which were evaluated using the applied scoring function. During the docking process, the key conformations of the protein were kept constant, whereas the ligands were allowed to be flexible [57]. The docked complexes were further visualized using Maestro and PyMOL software [47], and 2D and 3D images were generated.

### 2.11. Molecular dynamic simulation studies

MD simulations were performed using Desmond 2020.1, from Schrödinger [51]. Two complexes, specifically MOL002212MAPK1 and MOL002211ESR1, were chosen for MD simulation analysis due to their higher binding affinity and immune modulation roles, respectively. The OPLS-2005 force field and explicit solvent model employing SPC water molecules within a 10 ×  10 ×  10 Å periodic boundary solvation box were used [61]. Preprocessing, optimization, and minimization of protein-ligand complexes were performed using a protein preparation wizard. The system was constructed using a system builder tool [57]. Equilibration and minimization in the NPT (Nuclear Non-Proliferation Treaty) ensemble for 12 ns. The temperature and pressure were maintained at 310 K and 1 atm, respectively, using the Nose–Hoover chain coupling scheme [62]. Long-range electrostatic interactions were calculated using the particle mesh Ewald method with a 9 Å coulomb interaction cut-off. The simulation employed a 2-fs time step and the Martyna–Tuckerman–Klein chain coupling scheme for pressure regulation [57,63]. A 1000 ns simulation run was completed to ensure the stability of the protein-ligand complexes, monitored through metrics such as root mean square deviation (RMSD), root mean square fluctuations (RMSF), interaction fractions, ligand-protein contacts, radius of gyration (rGyr), solvent-accessible surface area (SASA), and polar surface area (PSA) [64].

### 2.12. Molecular mechanics to determine free binding energies

Molecular mechanistic studies using the Generalized Born Solvent Accessibility model (MM-GBSA) were performed to calculate the free binding energies (ΔG_Bind_) of the complexes (MOL002212MAPK1 and MOL002211ESR1). Prime MM-GBSA from Schrodinger Suite 2023-1 was used for performing MM-GBSA in the present work, since it integrates the energies of molecular mechanics (EMM) with the optimized force field (OPLS_2005) potential for liquid simulations. The VGSB solvent model was used in the present work because of its capability to produce better solvent-free energy with the solvent generalized born model compared to the chloroform and solvation models [65]. The binding free energy (ΔG_Bind_) for specific proteins (MAPK1 and ESR1) and ligands (MOL002212 and MOL002211) was calculated using the formula,


$$\Delta G_{Bind}={\text{G}}_{\text{complex}}–\left({\text{G}}_{\text{protein}}+{\text{G}}_{\text{ligand}}\right)$$

where G_complex_ represents the energy of the receptor-ligand complex, G_protein_ is the energy of the receptor, and G_ligand_ is the energy of the unbound ligand.

## 3. Results

### 3.1. Identification of potential targets for phytochemicals in Kochiae Fructus

**Fig 2:**
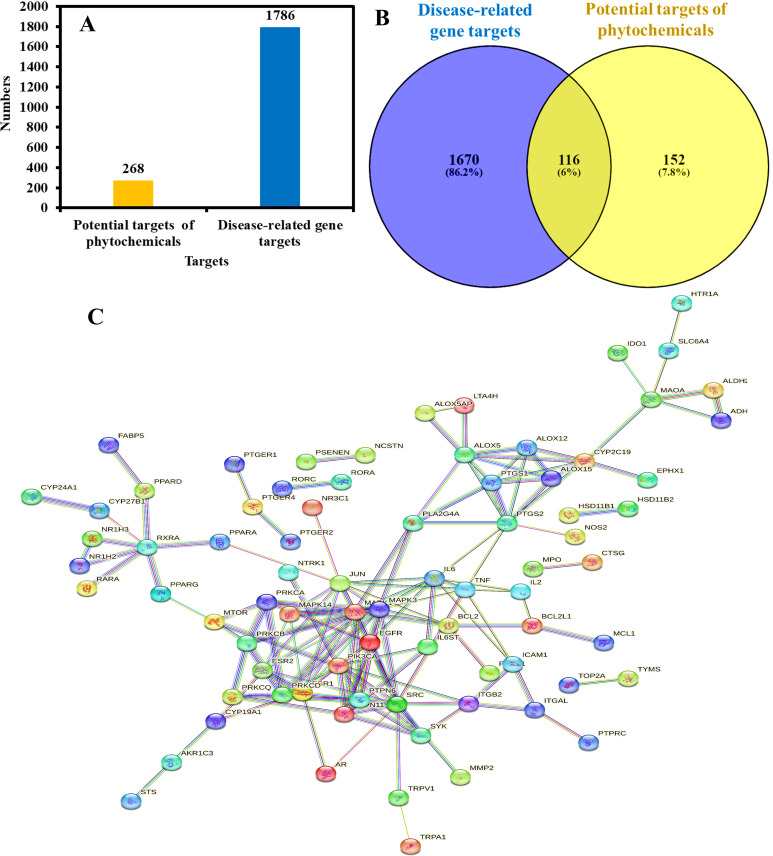
(A) Potential targets of Kochiae Fructus phytochemicals and AD-related gene targets. (B) Relationship between Kochiae Fructus phytochemicals and AD-related gene targets as well as their intersecting gene targets. (C) STRING protein-protein interaction (PPI) network of potential 78 anti-AD key targets of Kochiae Fructus.

Potential targets of Kochiae Fructus phytochemicals were acquired using the SwissTargetPrediction online database. A total of 1248 potential targets of 19 phytochemicals were identified, with a probability score of >  0. After eliminating redundancy, 268 potential targets were obtained and further analyzed (Fig 2A).

### 3.2. AD-related gene targets

A total of 3407 AD-related genes with a relevance score >  5 were obtained from the GeneCards database by searching the keywords “anti-atopic dermatitis,” “atopic dermatitis inhibiting,” and “atopic dermatitis.” After eliminating redundancy, 1786 gene targets were identified (Fig 2A) and investigated for further analysis.

### 3.3. Intersection gene targets analysis

The intersection gene targets were analyzed between the phytochemical potential targets and the AD-related genes using the Venny 2.1.0 web tool, and a total of 116 intersecting gene targets were identified and further analyzed (Fig 2B). Intersecting gene targets were considered as potential anti-AD key targets.

### 3.4. PPI network analysis of anti-AD key targets

The PPI network was constructed by loading potential 116 anti-AD key targets of Kochiae Fructus into the STRING database version 12.0, as shown in Fig 2C. The results demonstrated that the network comprised 78 nodes and 152 edges. Moreover, 38 nodes were disconnected and eliminated from the PPI network. The average node degree, average local clustering coefficient, and average PPI enrichment p-values were 2.62, 0.427, and *p* <  0.00001, respectively.

**Fig 3:**
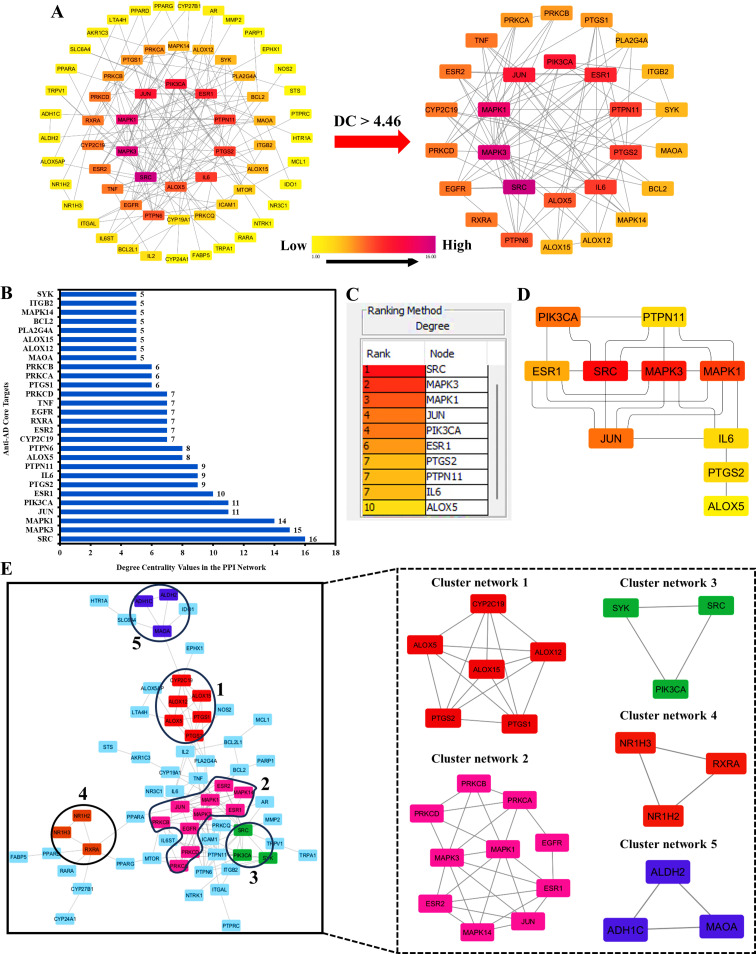
(A) Network maps of 65 and 28 potential anti-AD key and core targets (DC >  4.46) of Kochiae Fructus, respectively. (B) The bar graph presents 28 potential anti-AD core targets (DC >  4.46) with degree centrality values. (C) Ranking (D) Network map of the top 10 potential anti-AD core targets constructed using the CytoHubba plug-in in Cytoscape software. (E) MCODE cluster network analysis of the potential 78 anti-AD key targets. Cluster networks (1–5) were generated using the MCODE plug-in for Cytoscape.

As shown in Fig 3A, Cytoscape analysis results demonstrated that the PPI network had 78 nodes and 152 edges, with a characteristic path length of 3.962 between all node pairs. Furthermore, the density, diameter, average number of neighbors, clustering coefficient, network heterogeneity, network centralization, and network radius are 0.070, 10, 4.462, 0.346, 0.816, 0.186, and 5, respectively. Thirteen nodes appeared to be disconnected from the parent network and were therefore eliminated. Finally, the 65 nodes mapped in the network were arranged based on their DC values. The color of each node changed from yellow to purple as the degree increased.

The 28 nodes that complied with the criterion of DC>  average value (4.46) were further extracted and designated as potential anti-AD core targets of Kochiae Fructus (Fig 3A). The 28 potential anti-AD core targets ranked by their DC values are shown in Fig 3 (B) as a bar graph. Based on their DC values in the network, the top ten potential anti-AD core targets were SRC, MAPK3, MAPK1, JUN, PIK3CA, ESR1, PTGS2, PTPN11, IL-6, and ALOX5. The potential anti-AD core targets were further verified using the CytoHubba plug-in Cytoscape software, which ranked the same top eight targets based on their DC value in the network, as shown in Fig 3 (C) and 3 (D). Consequently, the top ten potential anti-AD core targets were chosen for molecular docking studies with the phytochemicals of Kochiae Fructus.

### 3.5. MCODE cluster analysis of the PPI network of anti-AD key targets

The PPI network, encompassing 78 potential anti-AD key targets, was subjected to an advanced cluster network analysis using the MCODE plug-in for Cytoscape. As depicted in Fig 3(E), this analysis revealed five distinct cluster networks within the anti-AD PPI landscape. Specifically, Cluster Network 1 comprised six nodes and 15 edges, achieving a score of 6, while Cluster Network 2 was characterized by ten nodes and twenty-six edges, yielding a score of 5.778. Conversely, Cluster Networks 3, 4, and 5 each manifested three nodes and three edges, each securing a score of 3.0.

Within Cluster Network 1, the nodes representing PTGS2, ALOX12, CYP2C19, PTGS1, ALOX5, and ALOX15 exhibited a high degree of interconnectivity with multiple gene targets, each attaining a DC value of 5.0. In the context of Cluster Network 2, the MAPK1, MAPK3, ESR1, PRKCA, MAPK14, JUN, ESR2, PRKCB, PRKCD, and EGFR nodes demonstrated extensive interconnectivity, with respective DC values of 8.0, 8.0, 6.0, 5.0, 5.0, 4.0, 4.0, and 2.0. Furthermore, in Cluster Network 3, PIK3CA, SYK, and SRC were interconnected with two additional gene targets, each with a DC value of 2.0. Similar connectivity patterns were observed in Clusters 4 and 5, where RXRA, NR1H2, and NR1H3, as well as ALDH2, ADH1C, and MAOA, were interconnected with two other gene targets, maintaining a DC value of 2.0, for each node.

Significantly, Cluster Networks 1, 2, and 3 corroborated the identification of the top eight quintessential anti-AD core targets, namely SRC, MAPK3, MAPK1, JUN, PIK3CA, ESR1, PTGS2, and ALOX5, as illustrated in Fig 3(E). This coherence between the cluster and PPI network analyses substantiates the robustness and validity of the identified anti-AD key targets, underscoring their potential therapeutic relevance in the context of atopic dermatitis.

### 3.6. Network of Kochiae Fructus’s phytochemicals and anti-AD targets

**Fig 4:**
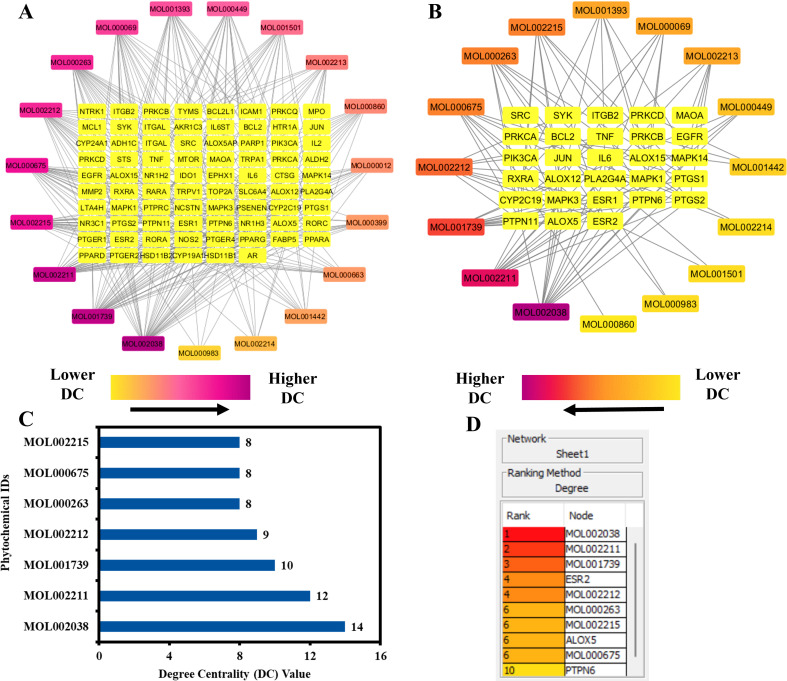
(A) Network map of 19 Kochiae Fructus phytochemicals and 78 potential anti-AD key targets. (B) Network map of 16 Kochiae Fructus phytochemicals and 28 potential anti-AD core targets. (C) The bar graph presents the top seven key phytochemicals of Kochiae Fructus ranked by their DC>  average value (6.3125). (D) Ranking of the top seven key phytochemicals of Kochiae Fructus using the CytoHubba plug-in in the Cytoscape software.

A network map elucidating the interactions between Kochiae Fructus phytochemicals and 78 potential anti-AD key targets was constructed using the Cytoscape software, as shown in Fig 4 (A). This structured network map comprises 97 nodes and 387 edges, manifesting a network diameter and radius of four and two, respectively. The topological parameters included a network density of 0.079, heterogeneity coefficient of 1.088, and centralization index of 0.302. Additionally, network map analysis revealed an average number of neighbors of 7.567, a characteristic path length of 2.881, a clustering coefficient of 0.000, and a single connected component. Each edge within this network represents a biochemical interaction between the phytochemicals of Kochiae Fructus and the delineated anti-AD key targets. The degree of a node reflects its connectivity within the network, with the nodal color gradient transitioning from orange to purple to indicate varying degrees of node centrality, thus providing insights into the network’s structural complexity.

Fig 4(B) presents a hub network map delineated using Cytoscape, illustrating the molecular interactions between Kochiae Fructus phytochemicals and 28 potential anti-AD core targets (determined in Section 3.4). This intricate hub network map consisted of 44 nodes and 101 edges with network diameters, radii, and densities of 6, 4, and 0.144, respectively. Analysis of the hub network map demonstrated that all 16 Kochiae Fructus phytochemicals interacted with the 28 potential anti-AD core targets to varying degrees. Notably, MOL002038 (9E,12Z-octadecadienoic acid), MOL002211 (11,14-eicosadienoic acid), MOL001739 (Zoomaric acid), MOL002212 (Ecdysterone), MOL000263 (Oleanolic acid), MOL000675 (Oleic acid), and MOL002215 (Oleanic acid) are the top seven key phytochemicals, ranked by their DC>  average value (6.3125), that interact with more than six potential anti-AD core targets in the hub network. These phytochemicals are acknowledged as key phytochemicals in Kochiae Fructus and are depicted in the form of a bar graph ranked by the DC in the hub network (Fig 4C). Further validation using the CytoHubba plugin in Cytoscape corroborated the prominence of these seven phytochemicals based on their DC values (Fig 4 (D)). Consequently, our results demonstrate that the synergistic mode of interaction exists between the multiple key phytochemicals present in Kochiae Fructus and multiple anti-AD core targets in the therapeutic milieu of AD.

### 3.7. GO enrichment analysis of Kochiae Fructus’s phytochemicals in the treatment of atopic dermatitis

**Fig 5:**
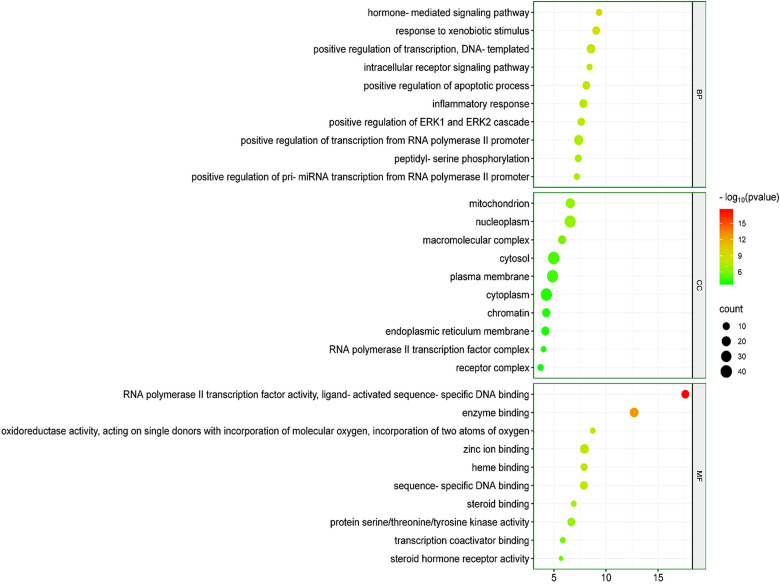
Top 10 GO enrichment analyses of 78 potential anti-AD key targets of Kochiae Fructus phytochemicals in the therapeutic milieu of AD.

The 78 potential anti-AD key targets implicated in AD were meticulously subjected to GO enrichment analysis. As depicted in Fig 5, the outcomes enumerate the top 10 enrichment terms across the molecular function (MF), cellular component (CC), and biological process (BP) categories pertinent to these targets. This analysis revealed that the anti-AD key targets of Kochiae Fructus’s phytochemicals within the BP category are intricately involved in an array of physiological pathways, including the hormone-mediated signaling pathway, response to xenobiotic stimuli, positive regulation of transcription, DNA-templated, intracellular signaling pathway, positive regulation of apoptotic response, inflammatory response, positive regulation of ERK1, and ERK2 cascade, etc. In the realm of CC, the analysis delineated that anti-AD key targets of Kochiae Fructus’s phytochemicals are predominantly localized within critical cellular structures such as the mitochondrion, nucleoplasm, cytosol, plasma membrane, cytoplasm, endoplasmic reticulum membrane, etc. This distribution suggests a comprehensive involvement of these anti-AD key targets of Kochiae Fructus phytochemicals in various cellular functions and processes that are pivotal in the pathophysiology of AD. Moreover, enrichment within the MF category was found to be dominated by functions such as RNA polymerase II transcription factor activity, ligand-activated sequence-specific DNA binding, enzyme binding, zinc ion binding, protein serine/threonine/tyrosine kinase activity, sequence-specific DNA binding, etc.

### 3.8. KEGG pathway analysis of Kochiae Fructus’s phytochemicals in the treatment of atopic dermatitis

**Fig 6:**
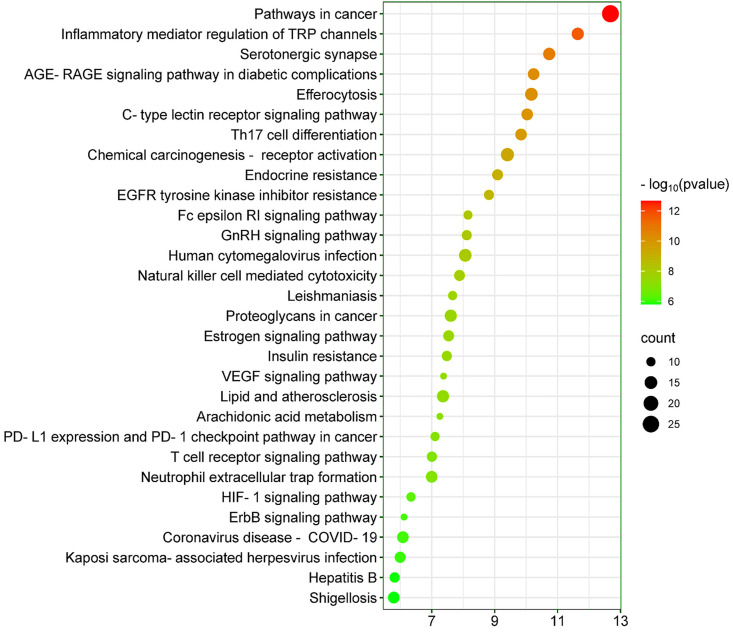
The top 30 KEGG pathways of 78 potential anti-AD key targets of Kochiae Fructus phytochemicals in the therapeutic milieu of AD.

KEGG pathway elucidation was performed to decipher the pharmacological mechanisms by which Kochiae Fructus phytochemicals exert ameliorative effects on AD. The uploading of 78 potential anti-AD key targets to the DAVID platform resulted in the identification of 138 statistically significant pathways (p <  0.05). Fig 6 shows the 30 most critical KEGG pathways ascertained through this analysis, which revealed that the anti-AD key targets of the Kochiae Fructus phytochemicals might be implicated in pathways in cancer, inflammatory mediator regulation of TRP channels, AGE-RAGE signaling pathway in diabetic complications, C-type Lectin Receptor Signaling Pathway, Th17 cell differentiation, chemical carcinogenesis-receptor activation, EGFR tyrosine kinase inhibitor resistance, proteoglycans in cancer, VEGF signaling pathway, lipid and atherosclerosis, T cell receptor signaling pathway, HIF-1 signaling pathway, and ErbB signaling pathway. This comprehensive analysis accentuates the complex and multifaceted pharmacological milieu wherein the phytochemicals of Kochiae Fructus ostensibly modulate a range of pathophysiological pathways that are implicated in AD. The elucidation of these pathways not only augments our understanding of the pharmacodynamic underpinnings of Kochiae Fructus’s therapeutic potential in AD treatment but also heralds the advent of innovative therapeutic modalities targeting these molecular pathways, thereby augmenting the precision and efficacy of AD management.

### 3.9. Core pathway analysis and determination

**Fig 7:**
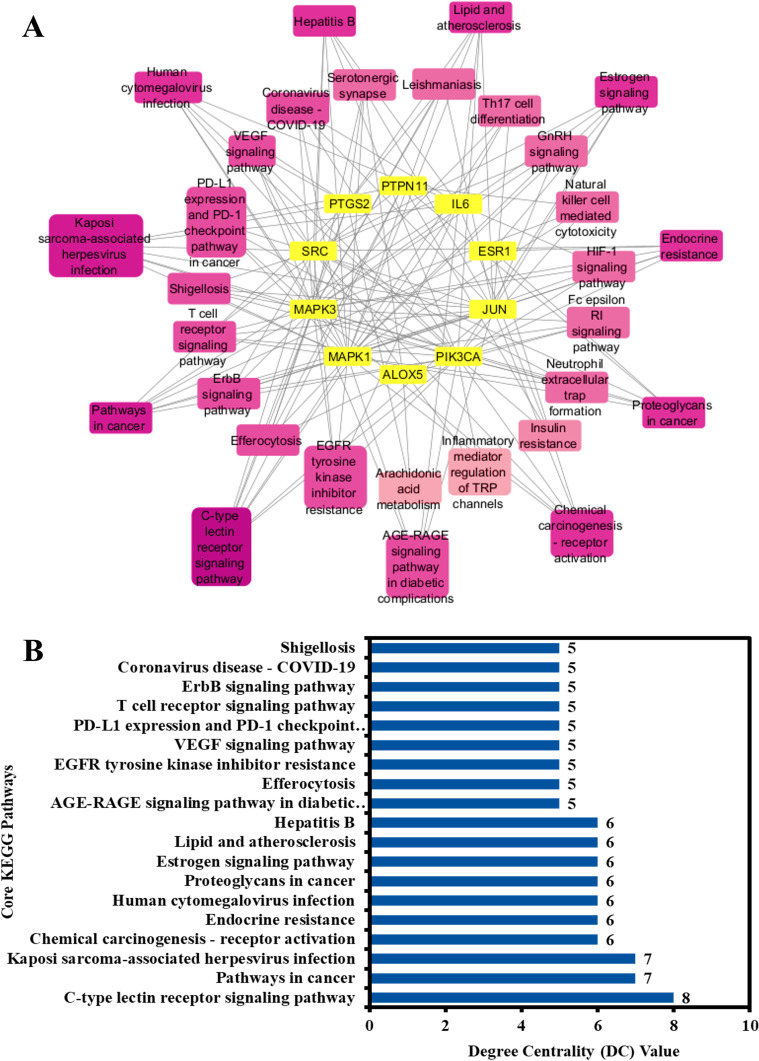
(A) Network map of the top ten anti-AD core targets and the top 30 KEGG pathways. The color of the pathway nodes changed from pink to light pink as the DC value decreased. (B) 19 core pathways with DC values (ranked by DC>  average value of (4.933)).

To elucidate the core pathways implicated in the anti-AD properties of the phytochemicals of Kochiae Fructus within the therapeutic milieu of atopic dermatitis, a network map was established by integrating the top ten anti-AD core targets and the top 30 KEGG pathways (Fig 7A). The results showed that the network map comprised 40 nodes and 148 edges, with a network diameter of 5, a radius of 3, and a density of 0.158. Network analysis indicated that all 30 pathways interacted with the ten anti-AD core targets at different DC values. The pathway with the highest degree (8.0) was the C-type lectin receptor signaling pathway, whereas arachidonic acid metabolism had the lowest degree (2.0). The color of the pathway nodes transitioned from pink to light pink depending on the degree.

Furthermore, the pathways in the network were ranked by DC>  average value of (4.933), resulting in the identification of 19 core pathways, as illustrated in Fig 7 (B). Eight of the ten anti-AD core targets (JUN, SRC, PTPN11, PTGS2, IL6, PIK3CA, MAPK1, and MAPK3) followed the C-type lectin receptor signaling pathway. In contrast, seven out of ten anti-AD core targets (PTGS2, MAPK1, MAPK3, JUN, ESR1, IL6, and PIK3CA) followed the pathways in cancer. Consequently, this study suggests that these 19 core pathways may play a role in the anti-AD effects of Kochiae Fructus phytochemicals.

### 3.10. Molecular docking analysis

**Fig 8:**
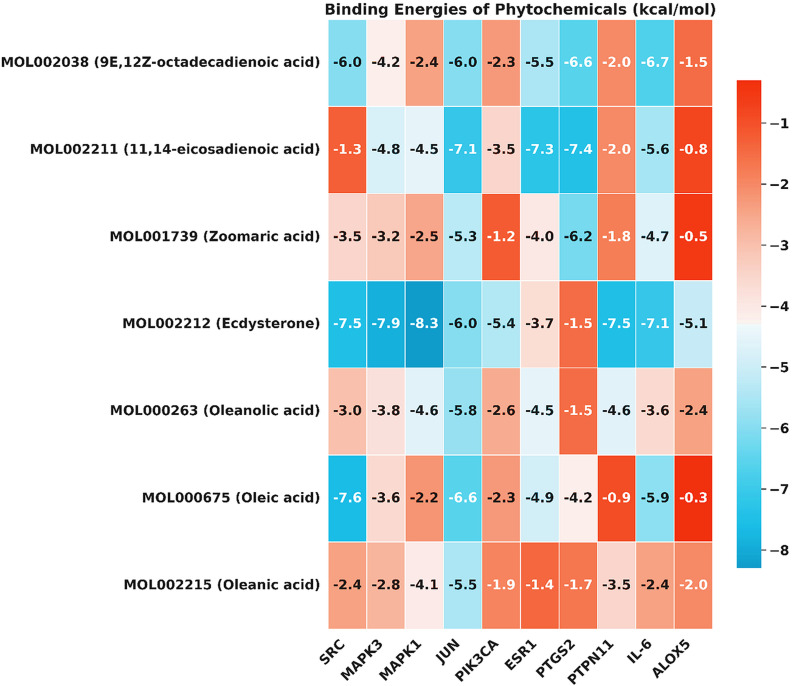
The heatmap exhibits the binding affinity (kcal/mol) scores for the top seven key phytochemicals of Kochiae Fructus, with the top ten anti-AD core targets. Each cell color indicates the strength of the binding energy, with cooler colors (blues) indicating stronger binding and warmer colors (reds) indicating weaker binding.

The top seven key phytochemicals of Kochiae Fructus were further analyzed for molecular docking with the top ten anti-AD core targets, including SRC, MAPK3, MAPK1, JUN, PIK3CA, ESR1, PTGS2, PTPN11, IL-6, and ALOX5. The results of the molecular docking analysis in terms of binding affinity (kcal/mol) scores are presented in Fig 8.

**Fig 9:**
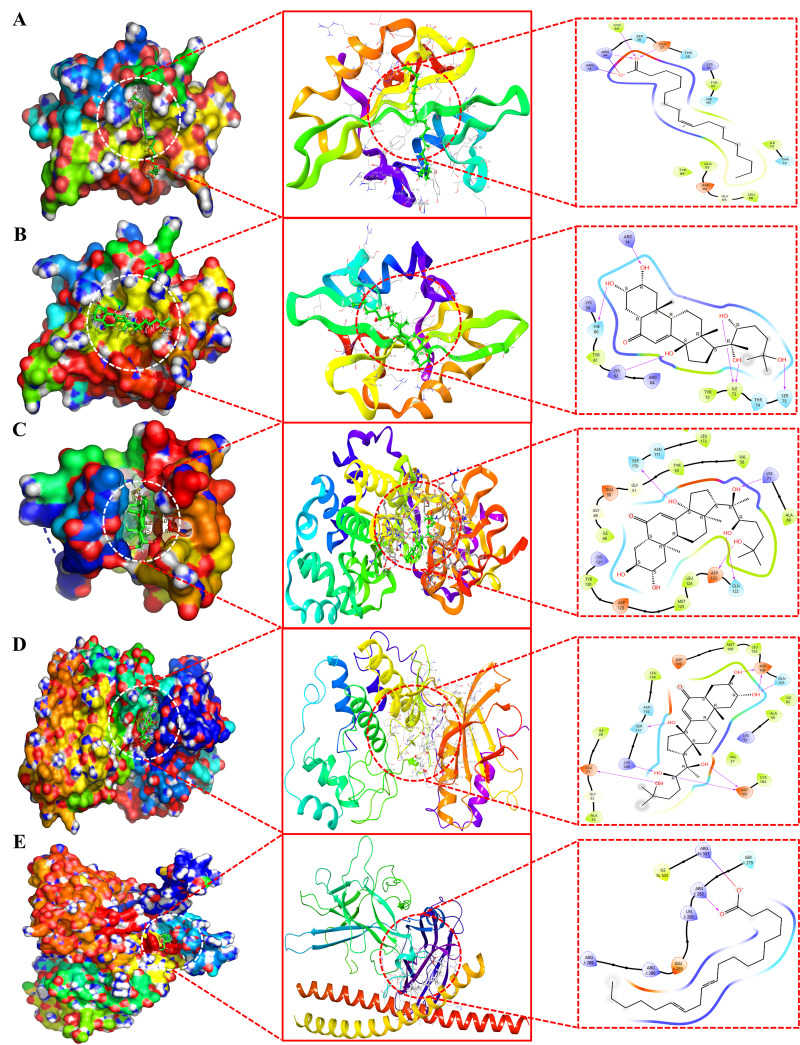
3D and 2D molecular docking results. (A) Oleic acid (MOL000675) and (B) ecdysterone (MOL002212) docked complexes with SRC. Ecdysterone (MOL002212) docked complex with (C) MAPK3 and (D) MAPK1. (E) 11,14-eicosadienoic acid (MOL002211) docked complex with JUN.

**Fig 10:**
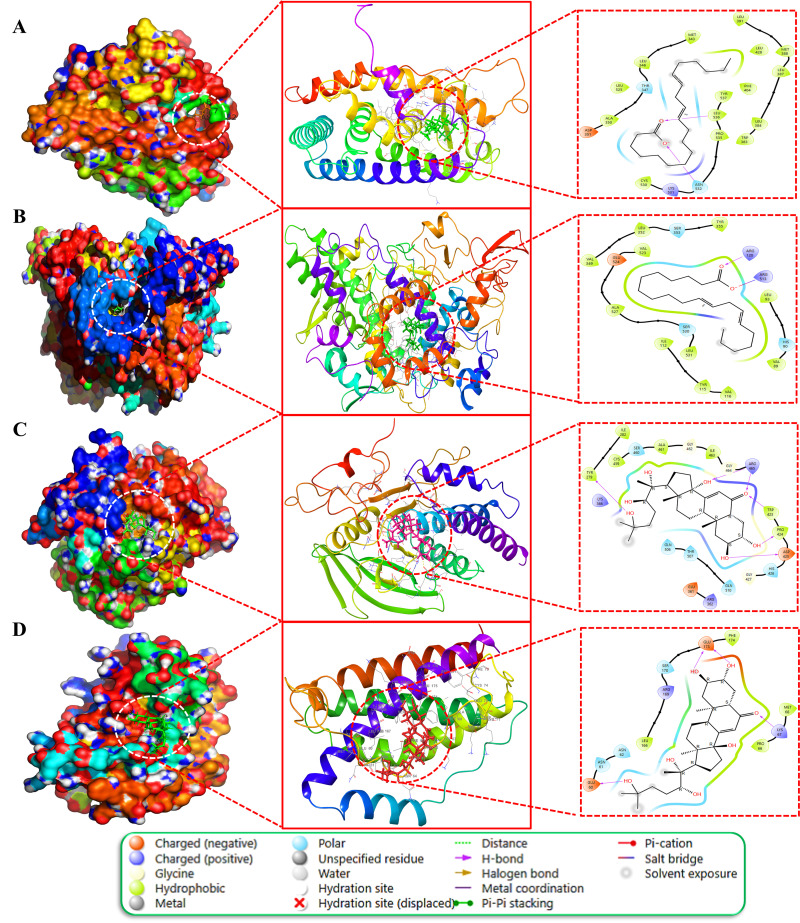
3D and 2D molecular docking results. 11,14-eicosadienoic acid (MOL002211) docked complex with (A) ESR1 and (B) PTGS2. Ecdysterone (MOL002212) docked complex with (C) PTPN11 and (D) IL-6.

Furthermore, the nine docked complexes, specifically MOL000675SRC, MOL002212SRC, MOL002212MAPK3, MOL002212MAPK1, MOL002212PTPN11, MOL002212IL6, MOL002211JUN, MOL002211ESR1, and MOL002211PTGS2, demonstrated binding affinities exceeding -7.0 kcal/mol. These complexes are depicted in both 2D and 3D formats in Fig 9 (A-E) and Fig 10 (A-D). Notably, compared to other phytochemicals, ecdysterone (MOL002212) exhibited exceptional binding affinities (-7.9 ± 0.04, -8.3 ± 0.03, -5.4 ± 0.03, -7.5 ± 0.02, -7.1 ± 0.04, and -5.1 ± 0.03 kcal/mol) for six anti-AD core targets including MAPK3 (H-Bond: LYS71, GLN122, ASP123, SER170), MAPK1 (H-Bond: GLU31, GLN103, ASP104, LYS149, SER151, ASP165), PIK3CA (H-Bond; HIE160, SER161, GLU218, LYS264, LYS290), PTPN11 (H-Bond: TYR279, TRP423, PRO424, ASP425, ARG465), IL-6 (H-Bond: GLU60, LYS67, GLU173), and ALOX5 (H-Bond: TRP147, ARG411), respectively. Conversely, 11,14-eicosadienoic acid (MOL002211) demonstrated notably high affinities (-7.1 ± 0.03, -7.3 ± 0.02, and -7.4 ± 0.02 kcal/mol) for JUN (H-Bond: ARG282; Salt bridge: ARG537), ESR1 (H-Bond: ASN532, LEU536), and PTGS2 (H-Bond: ARG120; Salt bridge: ARG513) respectively. Furthermore, oleic acid (MOL000675) displayed the highest binding affinity of -7.6 ± 0.03 kcal/mol against SRC and interacted with ARG34, GLU37, and CYS 44 through three hydrogen bond interactions and with ARG14, and ARG34 through two salt bridges. In conclusion, ecdysterone (MOL002212) and 11,14-eicosadienoic acid (MOL002211) demonstrated the best binding affinities with several anti-AD core targets compared with the other five key phytochemicals of Kochiae Fructus. Therefore, docked complexes of these two phytochemicals were further evaluated using MD simulations.

### 3.11. Molecular dynamic simulation analysis

**Fig 11:**
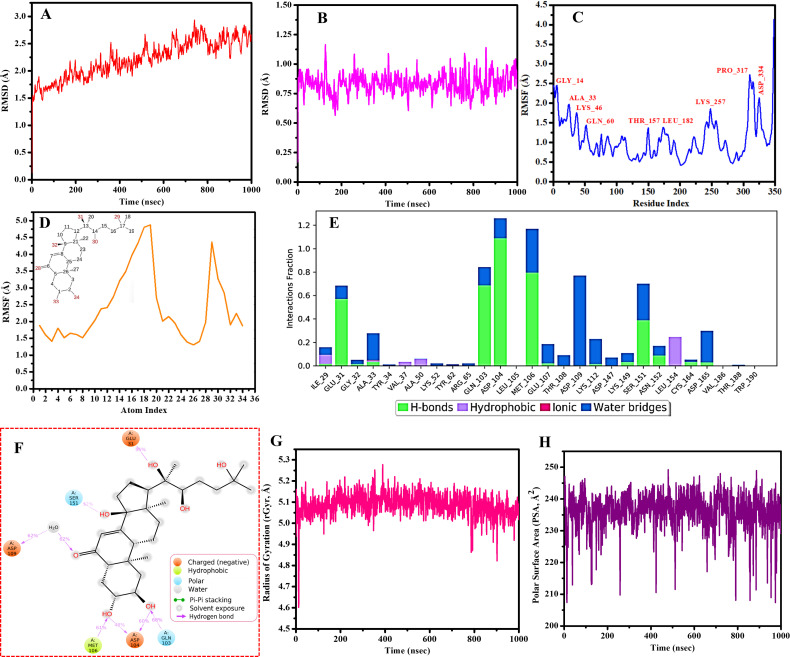
Molecular dynamics simulation results for the MOL002212MAPK1 complex. **(A)** RMSD of the Cα backbone of MAPK1 bound to ligand MOL002212. **(B)** RMSD of ligand MOL002212 bound to MAPK1. **(C)** Cα backbone RMSF of MAPK1 bound to the ligand MOL002212. **(D)** Ligand MOL002212 RMSF bound to MAPK1. **(E)** Interaction factions of amino acid residues MAPK1 with the ligand MOL002212. **(F)** Two-dimensional (2D) ligand-protein interaction diagrams (MOL002212MAPK1). **(G)** Radius of gyration (rGyr) and **(H)** polar surface area (PSA) of MOL002212 bound to MAPK1.

**Fig 12:**
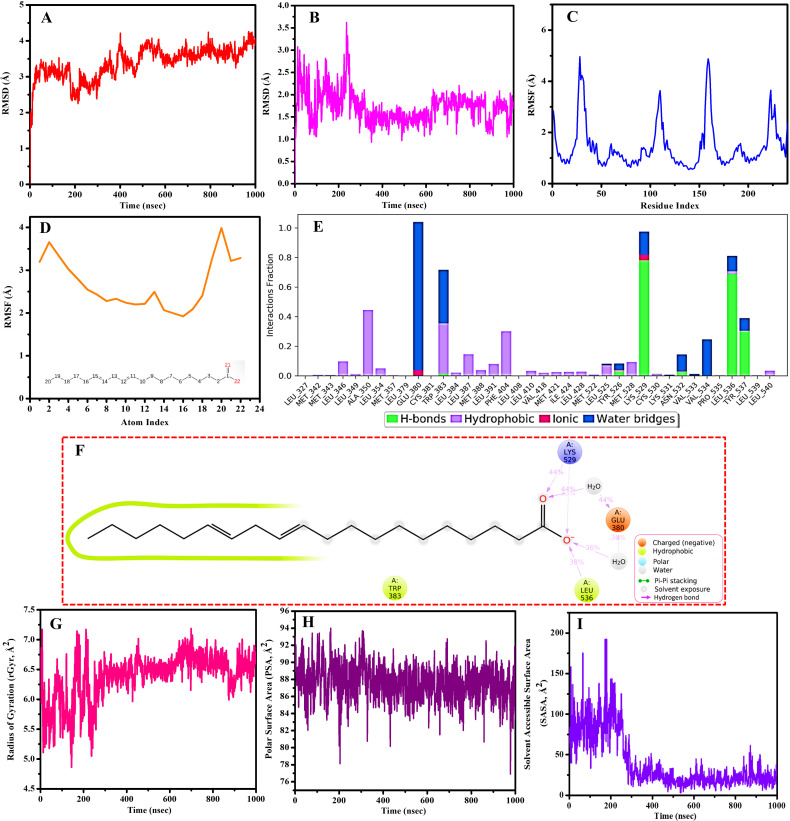
Molecular dynamics simulation results for the MOL002211ESR1 complex **(A)** Cα backbone RMSD of ESR1 bound to the ligand MOL002211. **(B)** RMSD of ligand MOL002211 bound to ESR1. **(C)** Cα backbone RMSF of ESR1 bound to ligand MOL002211. **(D)** Ligand MOL002211 RMSF bound to ESR1. **(E)** Interaction of amino acid residues of ESR1 with ligand MOL002211. **(F)** Two-dimensional (2D) ligand-protein interaction diagrams (MOL002211ESR1). **(G)** Radius of gyration (rGyr), **(H)** polar surface area (PSA), and (**I**) solvent-accessible surface area (SASA) of MOL002211 bound to ESR1.

Two complexes, specifically MOL002212MAPK1 and MOL002211ESR1, were chosen for the MD simulation analysis owing to the higher binding affinity and immune modulation roles, respectively. The results for each complex are shown in Fig 11(A-H) and 12 (A-I). Molecular dynamics simulations of the MOL002212MAPK1 and MOL002211ESR1 complexes over a 1000 ns period demonstrated consistent and stable molecular conformations, as substantiated by root-mean-square deviation (RMSD) analyses. For MOL002212MAPK1, the RMSD associated with the Cα backbone exhibited an ascending trend until approximately 800 ns, subsequently achieving pronounced stabilization that persisted throughout the remainder of the simulation interval, as shown in Fig 11(A). The observed RMSD values for the MOL002212MAPK1 complex ranged between 1.5 and 3 Å, conforming impeccably to the established thresholds for acceptability, while the ligand RMSD varied from roughly 0.50 to 1.20 Å, indicative of substantial stability (Fig 11B). For MOL002211ESR1, an increase in RMSD was discernible until approximately 500 ns, after which a heightened level of stability was attained until the conclusion of the 1000 ns period. The RMSD values for MOL002211ESR1 fell within the 2–4 Å range, consistent with acceptability standards [57], and the ligand RMSD ranged from approximately 1 to 3.75 Å, suggesting high stability (Fig 12A and 12B).

Variations in the protein root-mean-square fluctuation (RMSF) spanned from 0.5 to 2.75 Å for MOL002212MAPK1 and from approximately 0.75 to 5.0 Å for MOL002211ESR1, with both complexes experiencing numerous ligand interactions. Minimal fluctuations were primarily noted below the 1.5 Å level for MOL002212MAPK1 and below the 2.0 Å threshold for MOL002211ESR1, highlighting the relative stability of both complexes (Fig 11C and 12C). The spatial congruence between the ligands and proteins was assessed to be less than 5.0 Å for MOL002212MAPK1 and less than 4.0 Å for MOL002211ESR1, reflecting minimal alterations in ligand conformation throughout the simulations (Fig 11D and 12D).

Interaction analysis of both complexes revealed that hydrogen bonds and water bridges were predominant, with fewer hydrophobic and ionic interactions throughout the simulation. Notably, hydrogen bonds were critical for maintaining the binding conformation of both complexes. This was corroborated by two-dimensional (2D) ligand-protein interaction diagrams, which illustrated that significant residue interactions occurred upon binding. For MOL002212MAPK1, five amino acid residues (GLU31, GLN103, ASP104, MET106, and SER151) formed direct hydrogen bonds with small molecules, whereas ASP109 facilitated water-mediated hydrogen bonding, with hydrogen bond proportions of 55%, 68%, 108%, 61%, and 62%, respectively for the direct interactions (Fig 11F). For MOL002211ESR1, LYS529 and LEU536 formed direct hydrogen bonds, and GLU380 formed a water-mediated bond, with hydrogen bond proportions of 73% and 38% for the direct interactions (Fig 12F). Interestingly, coherence was observed in the results of residue interactions in the MD simulation analysis and molecular docking for both complexes.

Ligand properties such as the radius of gyration (rGyr), solvent accessible surface area (SASA), and polar surface area (PSA) further underscored the complex’s molecular dynamics, with MOL002212MAPK1 showing rGyr between 4.6 and 5.3 Å and PSA between 208 and 249 Å², along with SASA ranging from 167.03 to 333.02 Å² (Fig 11G, 11H, and S1 Fig). The fluctuations in PSA observed for MOL002212MAPK1 (Fig 11H) might be influenced by interactions with key residues such as GLU31, ASP104, GLN103, and ASP109, which form hydrogen bonds and water-mediated interactions with the ligand. While MOL002211ESR1 exhibited rGyr between approximately 4.75 and 7.25 Å and PSA between approximately 78 and 94 Å², along with SASA ranging from 1.50 to 190 Å² (Fig 12G, 12H, and 12I).

The SASA of MOL002211 bound to ESR1 (Fig 12I) revealed the dynamic conformational changes of the ligand–protein complex throughout the MD simulation. The marked fluctuations observed in the early phase (0–200 ns) signify structural rearrangements as the ligand establishes stable interactions with critical binding site residues. Such deviations are typical while the system adapts to achieve optimal binding. After 200 ns, the SASA values stabilize, indicating that the ligand is well-accommodated within the binding pocket and that the complex has reached a stable conformation. This stabilization underscores the adaptability of the ligand–protein interaction and confirms the reliability of the binding pose over the simulation period.

### 3.12. Molecular mechanics generalized Born surface area calculations

The stability of the MOL002212MAPK1 and MOL002211ESR1 complexes was further examined by determining their Gibbs binding energies (ΔG_Bind_). The results revealed that the stability of these complexes was primarily driven by Coulomb interactions (ΔG_Bind_Coulomb), van der Waals forces (ΔG_Bind_vdW), and lipophilic interactions (ΔG_Bind_Lipo), as shown in Table 2. In contrast, covalent interactions (ΔG_Bind_Covalent) and solvation-free energy contributions (ΔG_Bind_SolvGB) were identified as factors contributing to the instability of these complexes. Notably, both complexes (MOL002212MAPK1 and MOL002211ESR1) exhibited significantly higher binding free energies, highlighting their robust and stable protein-ligand interactions. This suggests that the ligands in these complexes form highly effective and long-lasting binding interactions with their respective targets.

**Table 2:** Binding free energies for the MOL002212MAPK1 and MOL002211ESR1 complexes were calculated using MM-GBSA.

Energies (kcal/mol)	Docked Complexes	
	MOL002212MAPK1	MOL002211ESR1
ΔG_Bind_	-38.43	-44.03
ΔG_Bind_Lipo	-13.84	-26.68
ΔG_Bind_vdW	-27.05	-43.77
ΔG_Bind_Coulomb	-36.98	-39.34
ΔG_Bind_Hbond	-3.40	-1.59
ΔG_Bind_SolvGB	11.95	14.72
ΔG_Bind_Covalent	4.89	3.39

## 4. Discussion

The current research work investigated the key phytochemicals and elucidated the underlying mechanisms of Kochiae Fructus in treating AD disease. In all, nineteen molecules were retrieved from TCMSP, and the top seven key phytochemicals ranked by DC ≥  average value of (6.3125) interacting with more than six anti-AD core targets include MOL002038 (9E,12Z-octadecadienoic acid), MOL002211 (11,14-eicosadienoic acid), MOL001739 (Zoomaric acid), MOL002212 (Ecdysterone), MOL000263 (Oleanolic acid), MOL000675 (Oleic acid), and MOL002215 (Oleanic acid). The network results also showed that single key phytochemicals may interact with multiple anti-AD core targets and that multiple key phytochemicals can also interact synergistically with a single anti-AD core target. Thus, our findings indicate a synergistic mode of interaction between multiple anti-AD core targets and multiple key phytochemicals in Kochiae Fructus for treating AD.

PPI network analysis identified several key genes involved in the etiology of AD, including SRC, MAPK3, MAPK1, JUN, PIK3CA (PI3K), ESR1, PTGS2, PTPN11, IL-6, and ALOX (Fig 3). Lu et al. found a positive correlation between SRC expression and inflammatory progression in AD, suggesting that SRC is a potential target for AD therapy [66]. Additionally, IL-17 has been implicated in activating the MAPKs and P38/ERK MAPK pathways, crucial in immunopathology, cellular proliferation, and inflammatory dermatological disorders [67–69]. The AP-1 complex, activated by JUN, drives inflammation, making c-Jun/AP-1 a viable target for managing psoriatic skin inflammation via immune modulation [70,71]. The PI3K/Akt signaling pathway, regulated by PIK3CA, is pivotal for various cellular functions, including metabolism, growth, and survival. In AD, the aberrant activation of this pathway in peripheral T cells induces T cell proliferation and cytokine release (IL-6 and IL-10), thereby contributing to the pathology of AD [72]. ESR1 (ERα) activation, as shown by Iwano et al., leads to IL-23 secretion from dendritic cells, exacerbating inflammatory and psoriasis-like dermatitis in a mouse model [73]. Moreover, Niu et al. reported that ESR1 enhances the Th2-immune response and Th2 cytokine levels in AD mouse models [74]. Recent studies have revealed that achieving an equilibrium between IL-6 and IL-17 is crucial for bolstering the anti-inflammatory properties of IL-4. Upregulation of IL-6 stimulates IL-4 production, leading to the acute phase of AD and pain. Furthermore, they promote the proliferation of T-cells and B cells, resulting in the formation of Th-17 cells. Elevated IL-4 expression suppresses IgE production, causing induced skin inflammation [75–78]. In contrast, PTGS2 plays a role in maintaining homeostasis but has also been implicated in various clinical pathologies, including pain and inflammation [79,80]. Although the implication of PTPN11 in AD remains unclear, Wang et al. showed that PTPN11 (SHP2) activation by Trichomide A has posed immunosuppressive effects on activated T lymphocytes, thereby mitigating contact dermatitis [81]. ALOX5, a member of the lipoxygenase enzyme family, is implicated in converting polyunsaturated fatty acids into leukotrienes, is critical in allergic inflammatory responses, and is notably active in AD-related inflammation [82]. Hence, this study suggests that modulating the expression levels of these ten anti-AD core targets could mitigate inflammation and ameliorate the symptoms associated with AD.

GO enrichment analysis showed that the ameliorative effects of Kochiae Fructus phytochemicals on AD might be attributed to multiple gene targets implicated in multiple BP, such as positive regulation of apoptotic response, inflammatory response, hormone-mediated signaling pathway, response to xenobiotic stimuli, positive regulation of transcription, DNA-templated, intracellular signaling pathway, positive regulation of ERK1, and ERK2 cascade, etc. Reports demonstrate that inflammation and apoptosis significantly contribute to the pathogenesis of AD. Patients afflicted with AD demonstrate elevated Fas and FasL expression, which are integral components of the apoptosis pathway. Moreover, induction of apoptosis in keratinocytes triggers AD progression, leading to cellular death and compromising the integrity of the skin barrier. Furthermore, the exacerbation of apoptosis is potentiated by proinflammatory cytokines such as TNF-α and IFN-γ via the Fas/FasL pathway [83,84]. Hormone-mediated signaling pathways have been shown to profoundly influence the pathogenesis and clinical manifestation of AD, implicating stress responses and immune modulation in their effects. Insights into these pathways are pivotal for understanding AD mechanisms and developing targeted therapeutic interventions [85,86]. The ERK1 and ERK2 cascades integral to the mitogen-activated protein kinase (MAPK) pathway have been implicated in the generation of inflammatory cytokines in epidermal keratinocytes, which promotes the recruitment of immune cells such as T cells and dendritic cells, thereby intensifying the inflammatory milieu characteristic of AD [87]. Consequently, therapeutic strategies aimed at modulating the ERK1/2 cascade are viable approaches to mitigating AD. The anti-AD effects of Kochiae Fructus phytochemicals are presumably linked to an array of gene targets predominantly located in cellular compartments such as the mitochondrion, nucleoplasm, cytosol, plasma membrane, cytoplasm, and endoplasmic reticulum membrane. Enriched MF ontology analysis further reveals that these gene targets engage in diverse functions, encompassing RNA polymerase II transcription factor activity, ligand-activated sequence-specific DNA binding, enzyme binding, zinc ion binding, protein serine/threonine/tyrosine kinase activity, sequence-specific DNA binding, etc.

KEGG pathway enrichment analysis revealed that the anti-AD effects of the Kochiae Fructus phytochemicals could be associated with pathways in cancer, inflammatory mediator regulation of TRP channels, AGE-RAGE signaling pathway in diabetic complications, C-type lectin receptor signaling pathway, Th17 cell differentiation, EGFR tyrosine kinase inhibitor resistance, VEGF signaling pathway, lipid and atherosclerosis, T cell receptor signaling pathway, HIF-1 signaling pathway, and ErbB signaling pathway. Moreover, network analysis of the top 30 KEGG pathways and top ten anti-AD core targets identified 19 core pathways ranked by DC ≥  average value of (4.933), as shown in Fig 7 (B). Among them, C-type lectin receptor signaling pathway, pathways in cancer, Endocrine resistance, Estrogen signaling pathway, EGFR tyrosine kinase inhibitor, VEGF signaling pathway, T cell receptor signaling pathway, and ErbB signaling pathway are the core pathways followed by eight of the ten anti-AD core targets (JUN, SRC, PTPN11, PTGS2, IL6, PIK3CA, MAPK1, and MAPK3), seven of ten anti-AD core targets (PTGS2, MAPK1, MAPK3, JUN, ESR1, IL6, and PIK3CA), six of ten anti-AD core targets JUN, PIK3CA, SRC, MAPK1, ESR1, and MAPK3), six of ten anti-AD core targets (JUN, PIK3CA, SRC, MAPK1, ESR1, and MAPK3), five of ten anti-AD core targets (IL6, PIK3CA, SRC, MAPK1, and MAPK3), five of ten anti-AD core targets (PIK3CA, SRC, MAPK1, PTGS2, and MAPK3), five of ten anti-AD core targets (JUN, PIK3CA, MAPK1, PTPN11, and MAPK3), and five of ten anti-AD core targets (JUN, PIK3CA, SRC, MAPK1, and MAPK3), respectively.

**Fig 13:**
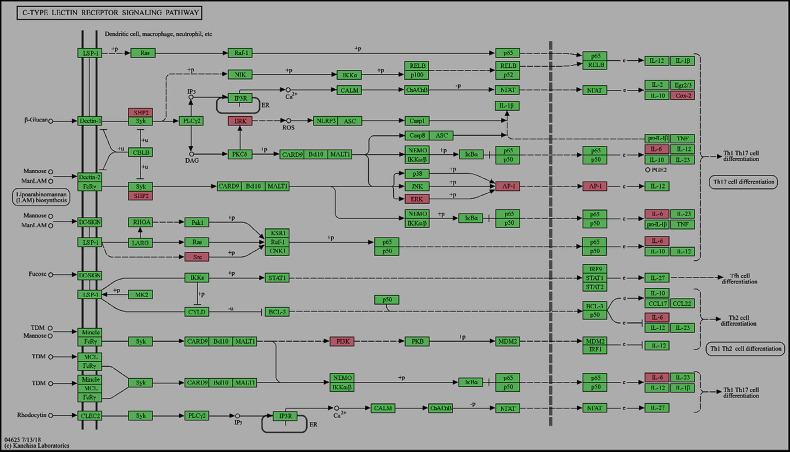
C-type lectin receptor signaling pathway (Pathway ID: hsa04625) from KEGG pathways. The diagram highlights the relevant pathway anti-AD core targets (SRC, SHP2 (PTPN11), ERK (MAPK1 and MAPK2), AP-1 (JUN), PI3K (PIK3CA), IL-6, and Cox-2 (PTGS2)) through a discernible pink hue.

Numerous studies have demonstrated the contribution of the C-type lectin receptor signaling pathway to AD pathogenesis, which exacerbates enhanced inflammatory (TNF-α, IL-6, and IL-1β) and Th2 immune responses [88]. Therefore, targeting the CLR signaling pathway may offer new therapeutic strategies for managing AD. The results showed that pathways in cancer regulation may contribute to the modulation of anti-AD targets. Targeting cancer-related pathways may also affect their expression in AD. Hyperactivation of the estrogen signaling pathway enhances the activities of Th2/regulatory T cells (Tregs) but suppresses Th1/Th17, leading to fortification of the skin permeability barrier [89]. Modulation of the estrogen signaling pathway may offer a promising approach for mitigating AD. Sismanopoulos et al reported that the VEGF signaling pathway facilitates the release of Vascular Endothelial Growth Factor from mast cells via IL-9 in AD, contributing to inflammation and vascular changes. Elevated IL-9 and its receptor expression in the affected skin underscores potential therapeutic targets in this pathway for disease management [90]. It has been demonstrated that the signaling pathways of T-cell receptors, in conjunction with the ligation of co-stimulatory molecules, facilitate T-cell-mediated inflammation. The inflammatory response plays a crucial role in the pathophysiology of AD [91,92]. Considering the well-established role of VEGF and T-cell receptor signaling pathways in the progression of AD, targeting these pathways presents a promising therapeutic approach for the disease. The ErbB receptor, from the tyrosine kinase family, includes ErbB1/EGFR/HER1 cell surface receptors that are integral to inflammation and AD, and primarily affect skin barrier integrity and immune response dynamics. Moreover, EGFR and MAPK are key targets of the ErbB signaling pathway. Dysregulation of this pathway exacerbates susceptibility to allergens and inflammatory reactions, contributing to the disease’s characteristic skin barrier dysfunction and heightened immune responses [93]. Therefore, the inhibition of the ErbB signaling pathway could serve as a potential therapeutic strategy for the treatment of AD. Hence, the results of the current study indicate that while several pathways may influence the anti-AD effects of Kochiae Fructus phytochemicals, the C-type lectin receptor signaling pathway is particularly significant. It not only interacts with a notably larger number of anti-AD core targets but also plays a more direct role in intensifying the crucial inflammatory and immune responses at the heart of AD pathogenesis (Fig 13). Consequently, the C-type lectin receptor signaling pathway is the most prominent pathway affected by Kochiae Fructus phytochemicals in the management of AD.

Our molecular docking study highlighted key phytochemicals from Kochiae Fructus, particularly ecdysterone (MOL002212) and 11,14-eicosadienoic acid (MOL002211), which exhibited significant binding affinities to anti-AD core targets. These findings suggest their potential use as therapeutic agents. Ecdysterone interactions, facilitated by extensive hydrogen bonding with targets, such as MAPK3 and MAPK1, suggest robust modulation capabilities against AD-related targets. Similarly, 11,14-eicosadienoic acid, supported by hydrogen bonds and salt bridges, displays high binding affinities to PTGS2, ESR1, and JUN, enhancing both stability and specificity. Moreover, it is noteworthy that both ecdysterone and 11,14-eicosadienoic acid exhibited strong binding affinities to all anti-AD core targets associated with the C-type lectin receptor signaling pathway, as shown in Fig 8 and 13.

MD simulations of the MOL002212–MAPK1 and MOL002211–ESR1 complexes confirmed stable interactions over 1000 ns, demonstrating robust conformational integrity throughout the simulation. By validating the docking predictions, these results underscore the resilience of each complex under physiological conditions, a key consideration for advancing therapeutic candidates. Prolonged stability *in silico* suggests these molecules may effectively bind their respective protein targets during extended exposure, increasing the likelihood of sustained pharmacological action. Such findings provide a solid foundation for subsequent *in vitro* and *in vivo* investigations aimed at assessing clinical viability and refining these agents for potential AD management. Moreover, Gibbs free energy (ΔG_Bind_) calculations underscored the spontaneity and stability of the binding interactions driven by favorable Coulombic interactions (ΔG_Bind_Coulomb), van der Waals forces (ΔG_Bind_vdW), and lipophilic interactions (ΔG_Bind_Lipo). This energetic analysis suggested that these complexes could exhibit favorable pharmacokinetic properties, which are essential for further therapeutic development.

Hence, this study advances traditional medicine research by showcasing how modern bioinformatics can uncover the therapeutic potential of Kochiae Fructus, a traditional herbal remedy for AD. Beyond validating its historical applications, the findings bridge the gap between traditional knowledge and contemporary drug discovery. By providing a robust, mechanistic framework, this work not only highlights the relevance of Kochiae Fructus in managing AD but also serves as a foundation for integrating other traditional medicinal herbs into modern therapeutic strategies. This integration marks a significant step forward in leveraging natural products for innovative, evidence-based therapies.

## 5. Conclusion

In this study, we systematically delineated the pharmacodynamic profile of Kochiae Fructus, a cornerstone of TCM, revealing its potential as a multifaceted therapeutic agent against AD. Utilizing state-of-the-art bioinformatics approaches, including network pharmacology and molecular docking, we identified 19 phytochemicals in Kochiae Fructus. Among these, seven phytochemicals demonstrated profound binding affinities to the top ten pharmacologically important anti-AD core targets, which were elucidated through robust PPI network analyses. Our findings underscore the profound impact of Kochiae Fructus on pivotal biological pathways that are integral to the pathophysiology of AD. GO and KEGG pathway enrichment analyses demonstrated that the anti-AD effects of Kochiae Fructus are intricately linked to the modulation of pathways, such as the C-type lectin receptor signaling pathway. This pathway’s engagement with multiple core anti-AD targets significantly influences inflammatory and immune processes central to AD pathogenesis, thereby shaping the scope of therapeutic approaches. Further substantiation from MD simulations and MM-GBSA calculations not only confirmed the stability and binding efficacy of these phytochemical-target interactions but also highlighted the potential of these compounds (ecdysterone and 1114-eicosadienoic acid) to act as potent modulators of AD pathology.

The results of this study provide a robust foundation for the therapeutic application of Kochiae Fructus in AD. By elucidating the molecular mechanisms and key targets, this study paves the way for the development of novel anti-AD agents derived from Kochiae Fructus, thereby contributing to more effective and targeted AD management strategies. These findings advance our understanding of the pharmacodynamic properties of Kochiae Fructus, including its target interactions, pathway modulation, binding affinity, and binding stability. The integration of network pharmacology with molecular docking and dynamic simulations offers a powerful framework for exploring the multifaceted interactions and therapeutic potential of traditional medicinal herbs in modern health care. Despite the comprehensive insights gained through network pharmacology and bioinformatics, the in-*silico* predictions presented here warrant experimental validation to confirm their efficacy, safety, and real-world applicability. Consequently, future *in-vitro* and *in-vivo* investigations—alongside clinical studies—are indispensable to substantiate and refine these findings for therapeutic application.

## Supporting information

S1 Fig Solvent-accessible surface area (SASA) of MOL002212 bound to MAPK1. (DOCX)

S1 Table List of phytochemicals identified with a probability score of >  0. (DOCX)

S2 Table Grid details of proteins used for molecular docking analysis. (DOCX)
