# Supplementary figures and images for: Network pharmacology and in silico analysis reveal Kochiae Fructus as a potential therapeutic against atopic dermatitis through immunomodulatory pathway interactions
Source: PLoS One. 2025 Apr 3;20(4):e0320818. doi: 10.1371/journal.pone.0320818 (PMC11967982; doi:10.1371/journal.pone.0320818)

**
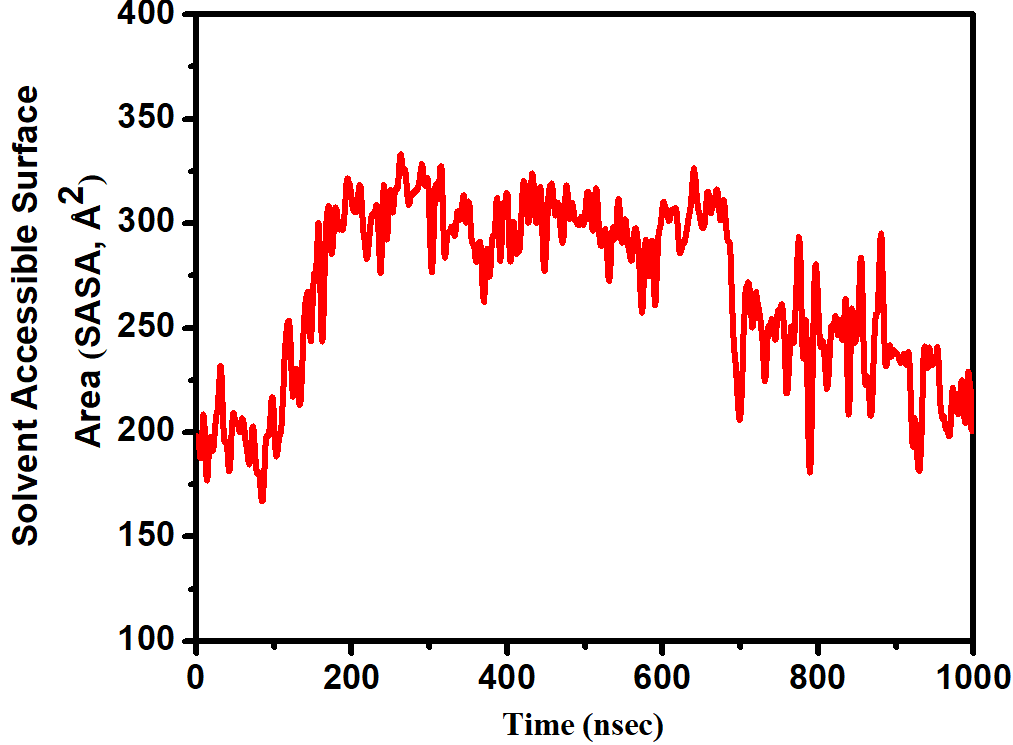
**

**S1 Fig.** Solvent-accessible surface area (SASA) of MOL002212 bound to MAPK1.

Supplement: S1 Fig — (DOCX) [file pone.0320818.s001.docx]
