# Supplementary material for: Network pharmacology and in silico analysis reveal Kochiae Fructus as a potential therapeutic against atopic dermatitis through immunomodulatory pathway interactions
Source: PLoS One. 2025 Apr 3;20(4):e0320818. doi: 10.1371/journal.pone.0320818 (PMC11967982; doi:10.1371/journal.pone.0320818)
Supplement: S1 Table — (DOCX) [file pone.0320818.s002.docx]

**S1 Table.** List of phytochemicals identified with a probability score of > 0.

| **Serial No** | **Phytochemicals ID** | **Phytochemicals Name** | **MW** | **OB (%)** | **DL** |
| --- | --- | --- | --- | --- | --- |
| 1 | MOL000012 | Arachic acid | 312.6 | 16.66 | 0.19 |
| 2 | MOL001393 | Myristic acid | 228.42 | 21.18 | 0.07 |
| 3 | MOL001442 | Phytol | 296.6 | 33.82 | 0.13 |
| 4 | MOL001501 | Daturic acid | 270.51 | 18.51 | 0.12 |
| 5 | MOL001739 | Zoomaric acid | 254.46 | 35.78 | 0.1 |
| 6 | MOL002038 | 9E,12Z-octadecadienoic acid | 280.5 | 41.9 | 0.14 |
| 7 | MOL002211 | 11,14-eicosadienoic acid | 308.56 | 39.99 | 0.2 |
| 8 | MOL002212 | Ecdysterone | 480.71 | 5.3 | 0.82 |
| 9 | MOL002213 | (Z)-1,3-di(phenyl)prop-2-en-1-one | 208.27 | 47.27 | 0.08 |
| 10 | MOL002214 | 96990-18-0 | 765.05 | 10.77 | 0.15 |
| 11 | MOL002215 | Oleanic acid | 440.78 | 8.41 | 0.77 |
| 12 | MOL000263 | Oleanolic acid | 456.78 | 29.02 | 0.76 |
| 13 | MOL000399 | Docosanoate | 340.66 | 15.69 | 0.26 |
| 14 | MOL000449 | Stigmasterol | 412.77 | 43.83 | 0.76 |
| 15 | MOL000663 | Lignoceric acid | 368.72 | 14.9 | 0.33 |
| 16 | MOL000675 | Oleic acid | 282.52 | 33.13 | 0.14 |
| 17 | MOL000069 | Palmitic acid | 256.48 | 19.3 | 0.1 |
| 18 | MOL000860 | Stearic acid | 284.54 | 17.83 | 0.14 |
| 19 | MOL000983 | n-Triacontanol | 438.92 | 10.46 | 0.45 |
