# Supplementary material for: Network pharmacology and in silico analysis reveal Kochiae Fructus as a potential therapeutic against atopic dermatitis through immunomodulatory pathway interactions
Source: PLoS One. 2025 Apr 3;20(4):e0320818. doi: 10.1371/journal.pone.0320818 (PMC11967982; doi:10.1371/journal.pone.0320818)
Supplement: S2 Table — (DOCX) [file pone.0320818.s003.docx]

**S2 Table.** Grid details of proteins used for molecular docking analysis.

| **Protein Name** | **PDB id of proteins** | **Grid details - inner box (X-Y-Z)** | **Grid details - outer box (X-Y-Z)** |
| --- | --- | --- | --- |
| SRC | 1O46 | 18, 22, 12 | 20.13, 22.72, 22.17 |
| MAPK3 | 4QTB | 22, 17, 15 | 36.94, 54.77, 50.23 |
| MAPK1 | 4FUX | 10, 10, 12 | 20.74, 6.47, 15.25 |
| JUN | 1A02 | 22, 23, 21 | 35.16, 40.38, 56.19 |
| PIK3CA | 6VO7 | 13, 14, 15 | 11.24, 21.17, -14.93 |
| ESR1 | 1XP1 | 14, 10, 10 | 31.49, -1.86, 24.27 |
| PTGS2 | 5KIR | 10, 10, 10 | 23.2, 1.34, 34.42 |
| PTPN11 | 4RDD | 10, 10, 10 | 27.84, 7.59, -0.22 |
| IL6 | 1IL6 | 19, 18, 10 | 0.84, 4.49, 7.93 |
| ALOX5 | 6NCF | 23, 18, 15 | 50.05, -24.26, -28.93 |
